# Cefiderocol in Combating Carbapenem-Resistant *Acinetobacter baumannii*: Action and Resistance

**DOI:** 10.3390/biomedicines12112532

**Published:** 2024-11-06

**Authors:** Bahman Yousefi, Setayesh Kashanipoor, Payman Mazaheri, Farnaz Alibabaei, Ali Babaeizad, Shima Asli, Sina Mohammadi, Amir Hosein Gorgin, Tahereh Alipour, Valentyn Oksenych, Majid Eslami

**Affiliations:** 1 Cancer Research Center, Faculty of Medicine, Semnan University of Medical Sciences, Semnan 35147-99442, Iran; 2 Student Research Committee, School of Medicine, Semnan University of Medical Sciences, Semnan 35147-99442, Iran; 3 Nervous System Stem Cell Research Center, Semnan University of Medical Sciences, Semnan 35147-99442, Iran; 4 Department of Clinical Science, University of Bergen, 5020 Bergen, Norway; 5 Department of bacteriology and Virology, Faculty of Medicine, Semnan University of Medical Sciences, Semnan 35147-99442, Iran

**Keywords:** *Acinetobacter baumannii*, β-lactamase, carbapenem, cefiderocol, PBP

## Abstract

*Acinetobacter baumannii* (*A. baumannii*) has emerged as a prominent multidrug-resistant (MDR) pathogen, significantly complicating treatment strategies due to its formidable resistance mechanisms, particularly against carbapenems. Reduced membrane permeability, active antibiotic efflux, and enzymatic hydrolysis via different β-lactamases are the main resistance mechanisms displayed by *A. baumannii*, and they are all effective against successful treatment approaches. This means that alternate treatment approaches, such as combination therapy that incorporates beta-lactams, β-lactamase inhibitors, and novel antibiotics like cefiderocol, must be investigated immediately. Cefiderocol, a new catechol-substituted siderophore cephalosporin, improves antibacterial activity by allowing for better bacterial membrane penetration. Due to its unique structure, cefiderocol can more efficiently target and destroy resistant bacteria by using iron transport systems. Through its inhibition of peptidoglycan formation through binding to penicillin-binding proteins (PBPs), cefiderocol avoids conventional resistance pathways and induces bacterial cell lysis. The possibility of resistance development due to β-lactamase synthesis and mutations in PBPs, however, emphasizes the need for continued investigation into cefiderocol’s efficacy in combination treatment regimes. Cefiderocol’s siderophore mimic mechanism is especially important in iron-limited conditions because it can use ferric-siderophore transporters to enter cells. Additionally, its passive diffusion through bacterial porins increases its intracellular concentrations, making it a good option for treating carbapenem-resistant *A. baumannii*, especially in cases of severe infections and ventilator-associated diseases (IVACs). Cefiderocol may reduce MDR infection morbidity and mortality when combined with customized antimicrobial treatments, but further investigation is needed to improve patient outcomes and address *A. baumannii* resistance issues.

## 1. Multidrug and Pandrug Resistant *Acinetobacter baumannii*

*Acinetobacter baumannii* (*A. baumannii*), a non-fermenting bacteria belonging to the *Moraxellaceae* family, has gained increasing prominence as a nosocomial pathogen in recent years. It was first discovered in 1911 by Beijerinck [1]. *A. baumannii* strains, which frequently display multidrug resistance (MDR), have become a significant clinical issue globally in the last decade. *A. baumannii* is frequently related to nosocomial infections including bacteremia, endocarditis, meningitis, urinary tract infections (UTIs), and gastrointestinal and skin or wound infections [2,3]. Antibiotic resistance in *A. baumannii* is often caused by a number of factors. One of these mechanisms is the inactivation of antibiotics by enzymatic hydrolysis, another is the reduction in membrane permeability, a third is the increase in antibiotic efflux, and a fourth is the mutation of binding antibiotic targets by genetic insertion sequences [4,5]. The majority of isolates that are resistant to carbapenem are also resistant to other antimicrobial classes, including aminoglycosides and fluoroquinolones. Additionally, some carbapenem-resistant endemic clones have sporadic polymyxin resistance. The rate of resistance to carbapenem varies between 40% and 80%, extensively drug-resistant (XDR) or pan drug resistant (PDR) is a common classification for these isolates [6]. Table 1 provides a comprehensive presentation of antibiotic classes, their mechanisms of action, resistance mechanisms, and the resistance status of *A. baumannii*.

## 2. Monotherapy and Combination Therapy of *Acinetobacter baumannii*

The amazing capacity of *A. baumannii* to acquire resistance against a wide range of medicines has made conventional treatment options increasingly troublesome for these infections. Because of this resistance, clinical care is severely limited and the infection-related morbidity and death rates are elevated. Treatment choices for *A. baumannii* infections are frequently influenced by variables such as the infection location, the patient’s general condition, and the isolated strain’s susceptibility profile [7]. The basic principle of treatment interventions against *A. baumannii* continues to be antibiotics; however, because of increasing resistance mechanisms, the range of medicines that are now accessible has decreased significantly. Penicillins and cephalosporins are among the beta-lactam antibiotics to which *A. baumannii* has previously shown sensitivity. But a lot of bacteria have become resistant to these antibiotics, mostly because they have acquired beta-lactamase enzymes, which hydrolyze them. The need to reevaluate conventional antibiotic therapy has arisen from the emergence of carbapenem-resistant *A. baumannii* (CRAB). Previously, the mainstay for treating severe *A. baumannii* infections was carbapenems, such as imipenem and meropenem; however, resistance to these antibiotics has been more common. The usage and study of more recent combinations of beta-lactam or beta-lactamase inhibitors have been prompted by this concerning trend [8].

In traditional methods, the limitations of polymyxin-based therapy are less considered due to their severe adverse effects, such as renal toxicity and dose limitations. Due to these problems, ampicillin-sulbactam or tigecycline, especially in high doses, is often recommended as the main treatment choice in the first stage of treatment. Because of their increased effectiveness, combination antimicrobial treatments are preferred over monotherapy [9]. Despite its nephrotoxicity, gentamicin is still a major agent and is often used in combination with other antimicrobials such as fosfomycin, tigecycline, ampicillin-sulbactam, or meropenem. The best treatment choice for significant CRAB infections is the combination of tigecycline, polymyxins, and high-dose ampicillin-sulbactam with additional antibiotics. Newer antibiotics like cefiderocol and durlobactam provide attractive therapeutic options, especially as backup plans in case the more established ones do not work [10].

The recent rise of MDR strains of *A. baumannii*, notably imipenem resistance, is one of the major issues associated with infection by this pathogen. *A. baumannii* infections caused by strains that are resistant to several drugs have an attributable mortality of 25–34%. Inappropriate treatment use is a factor associated with poor prognosis. Intrinsic and acquired resistance mechanisms strictly limit the antimicrobial choices for CRAB. The effectiveness and safety of antimicrobial drugs or combination regimens for the treatment of CRAB have been assessed in a few randomized studies. The majority of the data supporting various treatment approaches is observational or nonrandomized, has a small sample size, and has a wide range of illness severity and comorbidities [11].

Tigecycline with a high dose is preferred instead of the normal dose for CRAB, but this drug is not used alone when there are other antibiotic options [12]. Eravacycline has not been approved for the treatment of CRAB. Cefiderocol should only be used in conjunction with other medications to treat CRAB infections. But according to current studies, cefiderocol treatment for CRAB patients results in a greater death rate than treatment with the best treatment option (mostly polymyxin-based regimens) [13,14]. Intravenous fosfomycin-containing combination treatments for the treatment of CRAB have drawn more attention in recent years. However, there are insufficient data to support the use of this drug to treat life-threatening infections unless other options are available [13]. Some in vitro information recommends that the use of a long-infusion carbapenem as a third-line agent in combination with ampicillin-sulbactam and minocycline/polymyxin may result in bacterial eradication of CRAB. The usage of rifamycin for CRAB infections is constrained by certain clinical evidence, recognized toxicities, and drug–drug interactions [15,16]. Only ampicillin-sulbactam is currently thought to be suitable for monotherapy; all other medications should be taken in accordance with their susceptibilities in combination with ampicillin-sulbactam, with the exception of individuals who are allergic to penicillin. In the treatment of an infection like CRAB, the best choice is to use antibiotics. When an infection is related to high mortality, it is better to use antibiotic combination therapy. In infections where treatment options are limited, most physicians prefer to use combination therapy with agents that are separately active against a pathogen like CRAB. There are insufficient data to conclusively support any one single or combination treatment, despite numerous observational studies and randomized trials [11].

## 3. The Effect and Role of Beta-Lactamase Inhibitor on Cefiderocol

Cefiderocol is a pioneering antibiotic classified as a catechol-substituted siderophore cephalosporin, specifically engineered to combat infections caused by MDR Gram-negative bacteria (GNB), particularly strains resistant to carbapenems. Its innovative design includes an iron-chelating catechol moiety at the three-position side chain, which greatly improves its capacity to pass through GNB’s difficult outer barrier. Given the increasing threat posed by infections from organisms known for their resistance mechanisms, including Pseudomonas aeruginosa, carbapenem-resistant *Enterobacteriaceae*, and *A. baumannii*, this feature is especially significant [17]. Cefiderocol’s capacity to take advantage of bacterial iron transport systems is closely related to its mode of action. Cefiderocol exhibits strong chelating activity with ferric iron in an iron-limited environment, simulating the siderophores that bacteria naturally make to acquire iron. Iron is an essential component for the growth and survival of bacteria, which makes this action crucial. In contrast to other cephalosporins like ceftazidime, which do not have this ability, cefiderocol was demonstrated in experimental experiments to successfully chelate ferric iron. This distinction highlights how crucial the catechol moiety is to the structural makeup of cefiderocol, allowing it to function as a strong siderophore. Fluorescence-based assays using calcein were used to assess the transfer of iron into bacterial cells that cefiderocol enhanced. This technique is based on monitoring the fluorescence of intracellular calcein, which diminishes as it chelates with intracellular iron. The studies show that cefiderocol, like natural siderophores, actively stimulates the uptake of iron into *P. aeruginosa* PA01. Iron-regulated outer membrane proteins (IROMPs) are elevated in iron-depleted environments, which enhances this transport mechanism and makes it easier for cefiderocol to enter cells [18].

Cefiderocol has been shown to have strong in vivo activity against a variety of murine infection models, proving its antibacterial effectiveness beyond theory. Interestingly, it continues to work even against strains that show adaptive resistance to other antibiotics, indicating a clear benefit over current therapies. Cefiderocol has the potential to revolutionize the treatment of MDR infections due to the low rate of resistance development and the lack of cross-resistance with commercially available cephalosporins [19]. Chemically, cefiderocol is characterized by a four-membered β-lactam ring linked to a six-member dihydrothiazine ring, providing the structural foundation typical of β-lactam antibiotics. The C-3 location of the catechol moiety is essential because it enables the creation of stable chelate complexes with ferric iron, which facilitates iron transport across the bacterial cell wall and increases cefiderocol accumulation in the periplasmic region. In this instance, cefiderocol separates from the chelate complex and attaches itself to penicillin-binding proteins (PBPs), namely, PBP3, preventing the manufacture of peptidoglycans and ultimately causing the death of bacterial cells. Cefiderocol is different from other β-lactams because it uses a different method to penetrate bacteria. Cefiderocol uses its siderophore-like characteristics to actively interact with bacterial iron transport mechanisms rather than depending just on passive diffusion through porin channels. This increases its effectiveness against resistant strains of bacteria. In addition to circumventing known resistance mechanisms, this novel approach shows promise for creating analogous drugs that use this tactic to tackle the urgent problem of antibiotic resistance in Gram-negative bacteria [20].

**Figure 1:**
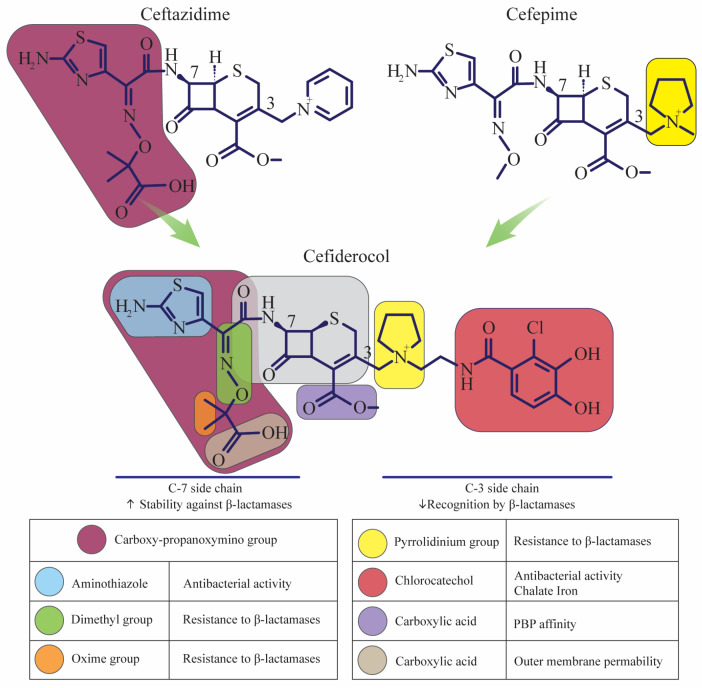
The structure of the cefiderocol antibiotic and the properties of its different parts. The structure analysis and characteristics of its main components are described below: 1. β-Lactam Ring: Structure: Like all cephalosporins, cefiderocol contains a four-membered β-lactam ring fused to a six-membered dihydrothiazine ring. The β-lactam ring is essential for the antibiotic’s bactericidal activity. It inhibits bacterial cell wall synthesis by binding to PBPs, preventing the cross-linking of peptidoglycan strands, which is crucial for cell wall integrity. This leads to bacterial cell lysis. 2. Catechol Group (Siderophore Moiety): Cefiderocol has a catechol group attached to its structure, which is responsible for its siderophore activity. The catechol group allows cefiderocol to chelate iron, a strategy bacteria use to survive in iron-limited environments. This mechanism helps cefiderocol to be actively transported into bacterial cells via iron transport systems. This siderophore activity gives cefiderocol an advantage in penetrating the outer membrane of GNB, especially those with efflux pumps or porin channel mutations. 3. Cephalosporin Core: Cefiderocol’s core structure is typical of cephalosporins but with modifications that enhance its ability to evade β-lactamase enzymes. This cephalosporin backbone gives cefiderocol its broad-spectrum activity by disrupting bacterial cell wall synthesis. It also provides stability against hydrolysis by various β-lactamases, including ESBLs and carbapenemases. 4. Side Chains: The chemical side chains attached to the core β-lactam structure modify the antibiotic’s pharmacokinetic properties, such as distribution and stability. The side chains in cefiderocol provide structural stability against β-lactamase enzymes, including metallo-β-lactamases, serine carbapenemases, and oxacillinases. These modifications enhance its effectiveness against carbapenem-resistant bacteria, which are often resistant to other β-lactams. 5. Carboxylic Acid Group: This negatively charged group is present in the cefiderocol structure. It helps the drug achieve good water solubility, which is important for intravenous administration and systemic distribution. Additionally, this feature helps in reducing renal toxicity [17]. (↑: Increase, ↓: Decrease).

β-lactamases have a significant effect on cefiderocol resistance, as demonstrated by increased cefiderocol activity when combined with inhibitors such as avibactam and dipicolinic acid. In isolates that express both MBLs and serine β-lactamases, this potentiation is more noticeable. Permeability flaws and enhanced efflux mechanisms also play a crucial part in resistance. Although some studies demonstrate different resistance levels due to changes in iron acquisition mechanisms, cefiderocol resistance has been linked to mutations in siderophore receptors, particularly *PiuA* and *PiuD* in *Pseudomonas aeruginosa*. *CirA* and *Fiu* mutations are common in *Enterobacterales*, particularly in New Delhi metallo-beta-lactamase (NDM). MBLs. Higher cefiderocol minimum inhibitory concentrations (MICs) are also caused by porin mutations (such as OmpK35 and OmpK36) in *P. aeruginosa* and *Klebsiella pneumoniae*. Cefiderocol MIC has also been linked to the upregulation of efflux pumps, such as MexAB–OprM in *P. aeruginosa* and AxyABM in *Achromobacter xylosoxidans*. When taken as a whole, these processes demonstrate the intricate interaction of genetic variables affecting cefiderocol resistance in diverse bacterial infections [21]. The structure of the cefiderocol antibiotic and the properties of its different parts are shown in Figure 1.

## 4. Mechanisms of Reduced Susceptibility to Cefiderocol

The most deadly opportunist pathogen is *A. baumannii*, which is also one of the most contagious [22,23]. Forming biofilms is one of the most significant virulence factors utilized by this MDR bacteria [23]. One of *A. baumannii*’s most important defense mechanisms against environmental and medicinal stressors is the formation of biofilms. When *A. baumannii* adheres to its substrate, it quickly aggregates into sessile biofilms. The extracellular matrix gives the biofilm its essential cohesive, hydrating, and viscoelastic characteristics. It is made up of polysaccharides, proteins, and nucleic acids. This dynamic growth mode promotes quorum sensing, horizontal gene transfer, hydration retention, and resistance to environmental stress [24]. *A. baumannii* biofilm growth mode on abiotic surfaces shields it from severe desiccation, allowing it to survive and perhaps foster epidemics. In addition to consuming external nutrients, electron donors, and acceptors, biofilms can also include dead and living host cells from other bacterial species. The evolution and growth of drug-resistant bacteria are facilitated by horizontal gene transfer, which happens more quickly in biofilms than in planktonic cells. This process is caused by the transfer of mobile genetic material such as plasmids containing antibiotic resistance genes [25]. Biofilms are important reservoirs for the spread of antimicrobial resistance (AMR) because exposure to sub-minimum inhibitory concentrations (sub-MICs) of antibiotics within the biofilm might further facilitate this gene transfer. Numerous investigations have demonstrated a positive relationship between *A. baumannii* biofilm production and AMR severity, with XDR strains creating more resilient biofilms than MDR strains [26]. Other results, however, indicate that XDR strains typically generate weaker biofilms compared to MDR and non-MDR bacteria. This disparity emphasizes how crucial it is to comprehend the co-regulatory mechanisms controlling AMR and biofilm development. The co-regulatory networks including genetic, environmental, and stress response factors that control biofilm development and AMR are probably complex. For instance, regulators like the BfmS/BfmR two-component system are known to control both biofilm formation and resistance traits in *A. baumannii*, suggesting overlapping regulatory networks. Additionally, biofilm formation can be influenced by environmental conditions, nutrient availability, and quorum sensing signals, which might vary between experimental conditions and clinical settings, contributing to the observed discrepancies [27].

The side chains of cefiderocol are essential to its penetration of the GNB outer membrane, which normally acts as a formidable barrier to antibiotic entrance. These changes increase the molecule’s hydrophilicity, which makes it more soluble in water and easier for it to diffuse via protein channels called porins, which let nutrients and tiny molecules pass through [28]. Cefiderocol’s ability to act as a chelator such as a siderophore is one of its unique characteristics. It utilizes the iron transport mechanisms of bacteria to enter cells through active transport systems and binds extracellular iron ions, which are necessary for bacterial development. Because of this absorption mechanism, cefiderocol can successfully target iron-dependent infections, especially in conditions where iron is restricted. When the cefiderocol-iron combination reaches the periplasmic region, it dissociates. PBPs, which are essential for the formation of bacterial cell walls, are then bound by cefiderocol. Cefiderocol inhibits PBPs, which prevents the synthesis of peptidoglycan, a crucial structural element of the bacterial cell wall, causing cell lysis and death [29,30]. Cefiderocol has exceptional stability against several metallo-β-lactamases, which are enzymes that impart resistance to other β-lactam antibiotics. However, there is growing in vitro evidence that metallo-β-lactamase-producing isolates can substantially impair cefiderocol’s efficacy against them, such as New Delhi metallo-beta-lactamase (NDM). This makes treating infections brought on by strains of bacteria that produce NDM difficult, emphasizing the need for continued research into combination treatments or new substances that might increase cefiderocol’s efficiency in these situations [31].

Apart from NDM β-lactamases, PER-like β-lactamase (PER-1) can hydrolyze cefiderocol and create resistance to this antibiotic [29,30]. Another study on *A. baumannii* clinical isolates showed that PBP-3 mutation can change cefiderocol MICs and cause resistance [32]. In a different study in Beijing, China, cefiderocol had promising results against carbapenem-resistant *Klebsiella pneumonia* and *P. aeruginosa,* while many CRAB isolates were resistant to cefiderocol. It was found that β-lactamase (PER) was active in all the cefiderocol non-susceptible CRAB isolates and was responsible for the resistance against this antibiotic [29]. It is predicted that β-lactamase inhibitors like Avibactam or Durlobactam can prevent PER-1-related hydrolysis of cefiderocol in non-susceptible *A. baumannii* isolates [33,34,35].

## 5. Mechanism of Action and Bacterial Resistance to Cefiderocol

Cefiderocol’s distinct mode of action and structural characteristics enable it to enter bacterial cells and circumvent several resistance mechanisms; however, resistance is still a major worry [31]. A detailed discussion of the processes of action and resistance follows.

### Mechanism of Action

Siderophore Activity: Cefiderocol binds to iron ions efficiently, which enables it to enter bacterial cells through active ferric-siderophore transport mechanisms. Since many viruses depend on iron scavenging for survival in iron-depleted environments, this mechanism is very helpful in those situations. Compared to other beta-lactams, Cefiderocol facilitates larger concentrations within the bacterial cell by imitating natural siderophores and improving their absorption.

Passive Diffusion through Porins: Like other beta-lactams, cefiderocol may also passively enter bacterial cells through porin channels in the outer membrane in addition to its siderophore-mediated absorption. Its efficiency against resistant organisms is enhanced by its dual route of entry, which guarantees that its intracellular concentrations are higher than those of conventional beta-lactams.

Release from Iron: Cefiderocol separates from the iron as soon as it reaches the periplasmic region. This is an important step because it releases the medication from its inactive state and enables it to continue its antibacterial action.

Resistance to Beta-Lactamase: The special structure of cefiderocol increases its stability against the inactivation of beta-lactam antibiotics by bacterial enzymes called beta-lactamases. Considering the prevalence of beta-lactamase-mediated resistance mechanisms, this resistance is a huge benefit.

Binding to PBPs: Penicillin-binding proteins, which are essential for the formation of bacterial cell walls, are efficiently bound by cefiderocol. It binds to these proteins and interferes with the formation of the cell wall, causing cell lysis and eventually bacterial death. Mutations in PBP-3 can compromise Cefiderocol’s binding affinity, ultimately reducing its ability to inhibit cell wall synthesis effectively. This alteration can lead to treatment failure in infections caused by resistant organisms.

**Figure 2:**
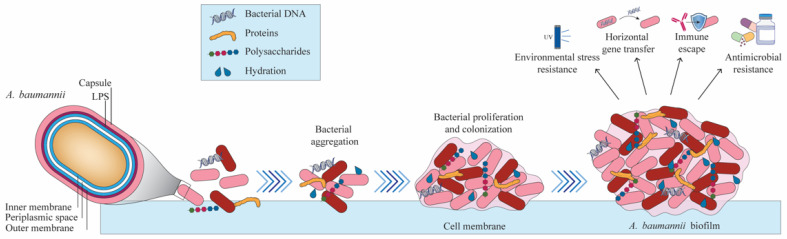
Biofilm formation in *A. baumannii* is a vital pathogenic mechanism that increases its survival and antibiotic resistance. This bacterium adheres to surfaces, forming biofilms that shield it from antimicrobial agents and the host immune system. Key genes like bap, csuE, and ompA play essential roles in biofilm development, promoting intercellular adhesion and structural integrity. Biofilms create a physical barrier and facilitate genetic exchange, aiding the spread of antibiotic resistance. As a result, biofilm-associated infections present significant challenges in clinical settings, particularly in hospitals.

**Figure 3:**
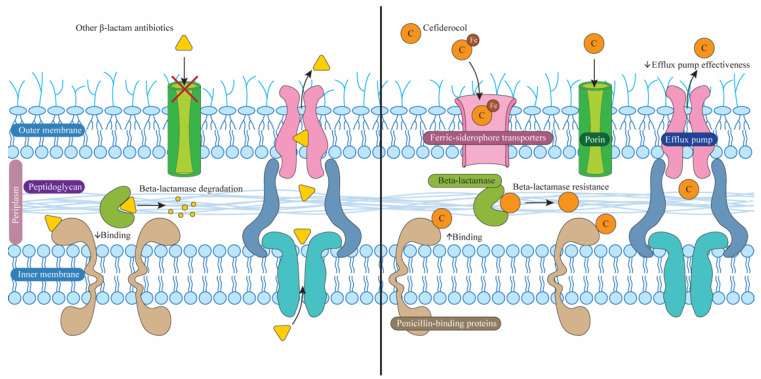
This figure highlights the differences in antibacterial effects between cefiderocol and other beta-lactams. Unlike most beta-lactams, cefiderocol can enter bacteria through ferric-siderophores and has a higher permeability via porins. Moreover, its increased affinity for PBPs and reduced efflux through efflux pumps contribute to its enhanced bacterial inhibition.

Resistance to Efflux Pumps: In comparison to other beta-lactams, cefiderocol is also more resilient to elimination by efflux pumps. Membrane proteins called efflux pumps aggressively remove antibiotics from bacterial cells, lowering the amounts of these drugs inside cells. Cefiderocol’s therapeutic efficacy against resistant strains is further enhanced by this feature [36,37], see Figure 2. The differences in antibacterial effects between cefiderocol and other beta-lactams shown in Figure 3.

## 6. Susceptibility Profiles Against Cefiderocol and Comparators

MDR bacteria, including carbapenem-resistant *Enterobacteriaceae*, CRAB, carbapenem-resistant *P. aeruginosa* (CR-PA), and MDR *Stenotrophomonas maltophilia* (MDR-SM), are considered super dangerous and life-threatening bacteria in healthcare systems [38]. Several new antibiotics have been created recently to treat Gram-negative, carbapenem-resistant bacteria. Cefiderocol is one of those that has the highest potential as an antibacterial agent. MIC values of cefiderocol against the different CRAB isolates ranged from 0.06 to >128 mg/L, with Minimum inhibitory concentration 50 (MIC50) and MIC90 values of 0.5 and 128 mg/L, respectively. Among the antimicrobial agents effective on CRAB, the highest susceptibility is related to colistin with 97.6% sensitivity, followed by cefiderocol and amikacin with 62.7% and 40.5% sensitivity, respectively. Comparing the data obtained from the cumulative percentage curves shows that colistin was the most active comparator agent [38]. Cefiderocol was employed as a rescue medication in a trial by Corcione et al. for acute respiratory distress syndrome brought on by CRAB in *Coronavirus* disease 2019 (COVID-19) patients. Cefiderocol was administered in combination treatment with colistin-sparing regimens for two-thirds of the patients. It is interesting that the total crude 30-day mortality was only 27.8%, compared to the 55% reported in a trial with a comparable cohort using cefiderocol monotherapy [39].

In different research by Falcone et al., 124 patients with CRAB-caused Ventilator-associated pneumonia (VAP) and blood infections were treated with cefiderocol in 37.9% of cases and colistin-containing regimens in 62.1% of cases. Patients receiving cefiderocol-containing treatment regimens experienced decreased 30-day mortality compared to those receiving colistin-containing regimens. As a result, the administration of cefiderocol in combination with colistin leads to a better prognosis, also colistin has high renal toxicity, and cefiderocol can be substituted for colistin in patients with kidney problems [38]. The administration of cefiderocol has been confirmed in the treatment of complicated urinary tract infections (cUTIs) caused by CRAB and the results of studies demonstrated the non-inferiority of cefiderocol to imipenem in the treatment of cUTIs [40]. Recently, the administration of cefiderocol has been recommended in hospital-acquired, ventilator-associated, or health-care-associated Gram-negative pneumonia caused by CRAB in conditions of resistance and intolerance to other drugs. However, cefiderocol is a first-choice medication for targeted therapy in critical patients due to its strong in vitro activity, high lung penetration, and anticipated safety profile [41].

In a phase 3 trial known as *Acinetobacter*, *Pseudomonas*, *Escherichia coli*, *Klebsiella*, *Stenotrophomonas* - nosocomial pneumonia (APEKS-NP), cefiderocol was also proven to be non-inferior to high-dose meropenem in patients with CRAB ventilator-associated pneumonia [42]. In a study by Po-Yu Liu et al., cefiderocol was the most active compound tested against CRAB, with MIC50/90 values of 0.5/2 mg/L compared to other extensively accessible antibiotics including ceftazidime (MIC50/90, >128/>128 mg/L), cefepime (MIC50/90,128/>128 mg/L), levofloxacin (MIC50/90, 16/128 mg/L), meropenem (MIC50/90, >64/>64 mg/L), ciprofloxacin (MIC50/90, >128/>128 mg/L), and amikacin (MIC50/90, >64/>64 mg/L) [43]. Most of the CRAB isolates that were sensitive to cefiderocol tested positive for *bla_OXA-23_* and *bla_TEM_*, whereas all of the isolates that were resistant to cefiderocol tested positive for *bla_PER_* genes as well as *bla_OXA-23_* and *bla_TEM_* [38].

## 7. Cefiderocol for Therapy in Carbapenem-Resistant *Acinetobacter baumannii*

Antibiotic resistance in GNB, which frequently results in longer hospital admissions, greater medical expenditures, and higher mortality rates, is one of the main global dangers to patients and healthcare systems. The final line of defense for treating infections brought on by MDR, ESBL-producing *Enterobacteriaceae*, is the β-lactam class of antibiotics known as carbapenems [44]. *Enterobacteriaceae*-producing carbapenemase and extended-spectrum β-lactamase, as well as *Pseudomonas* and *Acinetobacter*, are common MDR pathogens [45,46].

Carbapenem-resistant *Enterobacteriaceae*, carbapenem-resistant *P. aeruginosa*, and carbapenem-resistant *A. baumannii* were located in the main importance category by the global priority list of pathogens of the WHO in 2017 [47]. One of the important causes of hospital infection, especially in the ICU department, is *A. baumannii* [48]. This bacterium is responsible for HAP/VAP, bacteremia, as well as cUTI and ulcers, especially in ICU patients [49]. This bacterium is recognized by the WHO and Centers for Disease Control and Prevention (CDC) as a serious threat to public health. *Acinetobacter* has carbapenemase groups, the most common of which is Ambler’s D class, which consists of enzymes called oxacillinases (OXAs). Furthermore, Ambler’s class B, consisting of metallo-β-lactamases (NDM and Tripoli metallo-β-lactamase (TMB)) are also common [50].

Resistance to carbapenem in GNB is increasing, which limits treatment options and increases mortality caused by bacteria such as *Pseudomonas* and *Acinetobacter* [51,52]. In addition, carbapenems are the mainstay for treating infections caused by GNB [53,54]. Cefiderocol was approved in November 2019 by the FDA for treating cUTIs, including pyelonephritis, caused by susceptible GNB (*E. coli*, *K. pneumonia*, *Proteus mirabilis*, *Enterobacter cloacae* complex, and *P. aeruginosa*) and it was approved in September 2020 for a new indication, the treatment of HAP and VAP caused by *Enterobacterales*, *P. aeruginosa*, and *A. baumannii* complex [46,55,56]. It was also approved by the European Medicines Agency in 2020 for the treatment of infections caused by Gram-negative organisms resistant to multiple drugs [1,57]. Reaching this high concentration makes cefiderocol perform better than carbapenems, other cephalosporins, and β-lactamase inhibitors. It binds to PBPs and inhibits peptidoglycan cell wall biosynthesis. *Klebsiella pneumoniae* carbapenemase (KPC), NDM, and OXA are less sensitive to carbapenemases [58].

## 8. Cefiderocol and Its Implications in the Management of *A. baumannii* Infections

The reasons behind *A. baumannii* baseline resistance to cefiderocol, which is indicated by a MIC of less than 4 mg/L, have been studied by Simner et al. Permeability flaws and a mix of β-lactamase generation were among the main elements that their investigations uncovered. Interestingly, downregulation of the *pirA* gene and mutations in cell-wall-producing proteins, such as PBP3, have been connected to cefiderocol resistance in *A. baumannii*. Furthermore, through a variety of methods, contact with human blood or serum produces a hostile environment for *A. baumannii*, causing changes to its transcriptional profile and phenotype. It has been demonstrated that interactions between *A. baumannii* and Human Serum Albumin (HSA) and Human Pleural Fluid (HPF) control the expression of resistance and iron transporter genes. Studies conducted on the CRAB strain AB5057 revealed that the expression of the iron transporter was downregulated with exposure to HPF and 0.2% HSA. As CRAB strains AB0057, AMA16, and AB5075 were cultivated on LB medium supplemented with HPF, HSA, and human serum (HS), there was a notable decrease in the gene expression of transporters including bauA, pirA, and piuA as compared to LB media alone. Furthermore, it was discovered that the expression of genes linked to β-lactam resistance was elevated at physiological amounts of human serum (HS) [59,60]. An investigation into cefiderocol susceptibility was conducted due to worries about insufficient CSF concentrations, and the results showed susceptibility on day 8 (MIC 0.25 mg/L). CEF analysis revealed severe pleocytosis; hence, an IV regimen of cefiderocol 2 g every 6 h was selected. Day 10 saw a notable improvement in the patient’s condition, and the CSF cultures showed no signs of bacterial development [61].

**Figure 4:**
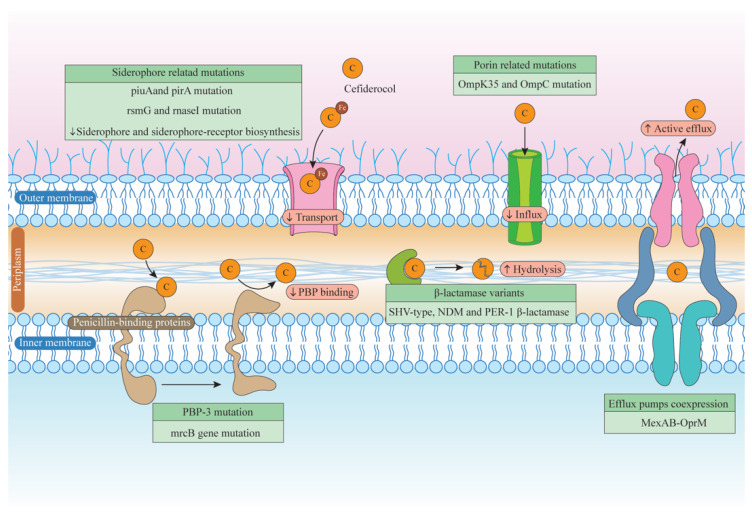
This figure illustrates various resistance mechanisms to cefiderocol, highlighting increasing evidence at each stage. Siderophore receptor mutations, such as piuA and pirA, impair active transport by decreasing siderophore biosynthesis. Additionally, mutations related to porin production can diminish passive influx. Moreover, the overexpression of efflux pumps facilitates the enhanced removal of cefiderocol. Resistance against certain β-lactamase variants is reduced, and mutations in PBP-3 can hinder cefiderocol’s binding capacity. Collectively, these factors contribute to increased resistance to cefiderocol. Up arrows (↑): export, down arrows (↓): import.

Cefiderocol was evaluated for concentrations in plasma and CSF on days 19 and 24, showing free maximum concentrations (fcmax) above the MIC and the *A. baumannii* breakpoint (4 mg/L). These findings support cefiderocol as a treatment option for CNS infections linked to *A. baumannii* [62]. Additionally, 20% of clinical isolates of *A. baumannii* have been connected to common or catheter-induced urinary tract infections (cUTIs). This indicates that *A. baumannii* can cause cUTIs. There has not been a lot of research conducted in this field; much of it has focused on lung and bloodstream infections [63]. To further investigate this phenomenon, Nishimura et al. conducted an experiment involving quantitative reverse transcription polymerase chain reaction (RT-PCR) assays, extracting total RNA from two CRAB strains (AB5075 and AMA40) cultured in iron-free cation-adjusted Mueller–Hinton broth (CAMHB) and CAMHB supplemented with 50% human urine (HU). The results indicated a marked increase in iron transporter expression in both strains cultured in CAMHB with HU. Additionally, changes in β-lactam gene expression were evaluated using qRT-PCR for pbp1, pbp3, blaOXA-23, blaOXA-51-like, blaADC, and blaNDM-1 in selected CRAB strains exposed to HU. Both strains showed increased expression of blaOXA-51-like, pbp1, and pbp3, and reduced expression of blaADC in AB5075 and blaNDM-1 in clinical isolate AMA40. The MIC values of CAMHB and CAMHB supplemented with 25%, 50%, and 100% HU were compared to ascertain susceptibility to cefiderocol. AB5075 showed a considerable drop in MIC, but AMA40 showed no discernible decrease. The results of this investigation might improve the use of cefiderocol and other antibiotics in the treatment of diseases that are resistant to several drugs, such as CRAB [64].The various resistance mechanisms to cefiderocol, highlighting increasing evidence at each stage shown in Figure 4.

## 9. Therapy of Infection-Related Ventilator-Associated Complications (IVACs)

An important problem in the care of patients in intensive care units (ICUs) requiring mechanical ventilation is IVACs. These side effects, which account for a large portion of hospital-acquired infections, raise the risk of serious illness, lengthen hospitalizations in the ICU, and increase medical expenses. IVACs complicate around one-third of hospital-acquired pneumonia (HAP) cases and have a very high mortality rate of more than 50%. The frequency of MDR and XDR infections, particularly GNB like *Pseudomonas aeruginosa* (*P. aeruginosa*) and *A. baumannii*, which are second in occurrence only to *Staphylococcus aureus*, is a major barrier to treating IVACs [65]. Empirical broad-spectrum antibiotic medication is usually the first step in the treatment of IVACs, and it is an important strategy for patients who are critically unwell and require prompt attention. But there are also a lot of hazards associated with this approach, especially if these antibiotics are overused, which might lead to the emergence of new resistance. When choosing an empirical therapy, it is important to take into account the epidemiological data specific to the area, especially when it comes to the frequency of resistant strains like *A. baumannii* that are resistant to carbapenem. After the causal organism has been microbiologically identified, it is critical to switch from broad-spectrum empirical therapy to tailored treatment that fits the susceptibility profile of the pathogen. Unfortunately, fewer than 40% of clinically diagnosed IVAC cases result in pathogen identification using standard culture methods, which can be delayed and take several days to give findings. This makes it more difficult to implement effective treatment plans [8].

IVACs make up to approximately 33% of HAPs, making it the most common mechanical ventilator-associated infection event in ICU patients [66]. Considering the high mortality rate of over 50% and high morbidity rate and treatment cost, IVAC is an important concern [67]. *A. baumannii* and *P. aeruginosa* are responsible for a vast majority of VAPs, second only to *Staphylococcus aureus* [68,69]. Due to the antimicrobial resistance development, treatment of these pathogens is a challenge [68], First-line therapy for CRAB-associated IVACs is 2 g Cefiderocol q8h continuous IV infusion after 2 g of the loading dose, and the second line is the combination therapy of fosfomycin (6–8 g loading dose (LD)) followed by 16–24 g/day continuous infusion (CI)), high-dose ampicillin-sulbactam (6 g/3 g q8h CI after 6–8 g/3–4 g LD), and inhaled colistin (2 million units (MU) q8h) [61]. A higher mortality rate in *A. baumannii*-associated IVAC was significant in patients receiving Cefiderocol treatment rather than the best available therapy (49% vs. 18%) [70]. Trecarich et al. reported using cefiderocol to successfully treat a VAP caused by CRAB [71].

In cases of pneumonia, including IVACs, recent developments in molecular diagnostic tools have greatly improved the speed and accuracy of pathogen identification. Rapid molecular testing is being incorporated more and more into clinical practice, enabling medical personnel to maximize antibiotic stewardship and make well-informed decisions about the administration of antibiotics. Therapeutic drug monitoring (TDM) and pharmacokinetics and pharmacodynamics (PK/PD) principles inform targeted antimicrobial therapy, which is essential for optimizing treatment effectiveness while reducing the likelihood of resistance development [72]. A multidisciplinary strategy comprising critical care physicians, infectious disease experts, clinical microbiologists, and clinical pharmacologists is necessary for the effective therapy of IVACs. By ensuring that antimicrobial medication is customized to each patient’s unique needs, this cooperative effort helps to reduce the overuse of antibiotics and improves clinical results. The increasing prevalence of MDR and XDR infections in intensive care units highlights the need for creating evidence-based IVAC treatment strategies. These algorithms are particularly useful for treating organisms that are challenging to treat, such as *P. aeruginosa* and *A. baumannii*. The use of new antimicrobial drugs is a skill that intensive care doctors need to possess, and antimicrobial supervision initiatives are crucial to maintaining the effectiveness of broad-spectrum antibiotics. The prognosis for patients with IVACs can be greatly improved by early and correct diagnosis along with focused therapy, which may lower the risk of infection and raise overall ICU care standards. In order to effectively manage IVACs for infections associated with *P. aeruginosa*, two key measures are necessary. First and foremost, it is imperative to use regimens that spare carbapenem [73,74].

Because of the increased risk of resistance associated with carbapenems, regimens based on piperacillin-tazobactam or third- and fourth-generation cephalosporins (such as ceftazidime or cefepime) are suggested for multi-susceptible isolates. Based on the unique resistance mechanisms of the strain, novel beta-lactam/beta-lactamase inhibitors (BL/BLIs) such as ceftolozane-tazobactam or ceftazidime-avibactam, as well as cefiderocol, should be taken into consideration for MDR/XDR isolates. Second, given the low pulmonary penetration of ceftazidime, cefepime, piperacillin-tazobactam, and cefiderocol, optimizing antibiotic dosing strategies—especially through large doses and extended infusions—is critical to achieving the required PK/PD objectives [75]. Due to toxicity and low PK/PD effectiveness, standard colistin or polymyxin-based regimens face substantial hurdles for MDR/XDR *A. baumannii*, which is linked to VAP and carries fatality rates as high as 60%. Cefiderocol should thus only be used in situations where there is evidence of intolerance or resistance to other active medicines. Nevertheless, cefiderocol is a top choice for MDR *A. baumannii* infections in critically sick patients due to its good safety profile, in vitro effectiveness, and ability to increase lung exposure with continuous infusion (CI) [76]. In combination therapy, fosfomycin has gained traction as a viable substitute, especially when combined with high-dose sulbactam as a second-line treatment for MDR *A. baumannii*. Studies have shown that patients who received fosfomycin-containing regimens had better survival rates when they had pneumonia, even when sulbactam was not part of the combination. It is suggested to use high-dose CI beta-lactam regimens to reach PK/PD goals of 100% fT > 4–8 times MIC in order to maximize therapeutic results. This will improve lung penetration and clinical effectiveness while reducing the development of resistance [77]. The Overview of IVACs and their Management is shown in Table 2.

## 10. Conclusions

Cefiderocol is an emerging therapeutic option for treating MDR *A. baumannii*, as traditional carbapenems like tigecycline (TGC) and Colistin (COL) are losing effectiveness due to rising resistance. This resistance results from mechanisms such as enzymatic inactivation by β-lactamases, drug efflux pump overexpression, and changes in antibiotic binding sites. Cefiderocol demonstrates significant in vitro activity against various Gram-negative bacteria, including *Pseudomonas aeruginosa* and CRE. It binds effectively to PBP-3 and shows resilience against hydrolysis by specific β-lactamases. Resistance in CRAB isolates is linked to the presence of bla_PER_, bla_OXA-23_, and blaTEM genes. Due to its efficacy, high lung penetration, and safety profile, cefiderocol is recommended for treating Gram-negative pneumonia caused by CRAB in critically ill patients.

## Figures and Tables

**Table 1:** Overview of Antibiotic Classes, Mechanisms of Action, Resistance Mechanisms, and Resistance Status in *A. baumannii*.

Antibiotic Class	Examples	Mechanism of Action	Resistance Mechanisms	Resistance Status
Carbapenems	Imipenem, Meropenem	Inhibit cell wall synthesis by binding to PBPs.	- Production of carbapenem-hydrolyzing enzymes (e.g., OXA-type). - Decreased membrane permeability.	High minimum inhibitory concentration (MIC) values in most isolates.
Cephalosporins	Ceftazidime, Cefepime	Inhibit cell wall synthesis via PBPs.	- Production of extended-spectrum beta-lactamase (ESBLs). - Hydrolysis of β-lactam ring.	Widespread resistance, particularly in third-generation.
Aminoglycosides	Amikacin, Gentamicin	Bind to bacterial ribosomes and inhibit protein synthesis.	- Production of aminoglycoside-modifying enzymes (AMEs). - Altered ribosomal target sites.	Over 72% resistance in isolates.
Polymyxins	Polymyxin B, Colistin	Disrupt the outer membrane of bacteria, leading to cell death.	- Modification of Lipopolysaccharides (LPS). - Reduced drug binding and uptake.	Resistance noted, but polymyxin B may show better efficacy.
Fluoroquinolones	Ciprofloxacin	Inhibit DNA replication by targeting DNA gyrase and topoisomerase IV.	- Mutations in gyrA and parC genes. - Overexpression of efflux pumps.	High resistance observed in many isolates.
Tetracyclines	Minocycline, Tigecycline	Inhibit protein synthesis by binding to the 30S ribosomal subunit.	- Efflux pumps. - Ribosomal protection proteins.	Minocycline is effective against all isolates; tigecycline variable.
Siderophore-Cephalosporins	Cefiderocol	Inhibits cell wall synthesis by binding to PBPs, utilizes bacterial iron transport mechanisms to enter cells	- Alterations in siderophore transport pathways. - Presence of certain β-lactamases (e.g., metallo-β-lactamases). - Efflux pump expression.	Resistance observed in some isolates.

**Table 2:** Overview of IVACs and their Management.

Aspect	Details
Prevalence of IVACs	Account for approximately 33% of HAPs; the most common mechanical ventilator-associated infection in critically ill patients.
Mortality Rate	Often exceeds 50%.
Associated Morbidity	Significant morbidity and increasing healthcare costs.
Common Pathogens	- *Acinetobacter baumannii* - *Pseudomonas aeruginosa* - *Staphylococcus aureus*
Challenges	Emergence of antimicrobial resistance complicates treatment options and increases adverse outcomes; resistant strains lead to severe clinical presentations and poorer prognoses.
First-Line Treatment for CRAB	Continuous intravenous infusion of cefiderocol: - Loading Dose: 2 g - Maintenance Dose: 2 g every 8 h
Mechanism of Action of Cefiderocol	Novel siderophore cephalosporin that binds to iron receptors on bacterial surfaces, enhancing penetration into bacterial cell walls.
Second-Line Treatment Options	- Fosfomycin: - Loading dose: 6–8 g - Continuous infusion: 16–24 g/day - High-Dose Ampicillin-Sulbactam: - 6 g/3 g every 8 h via continuous infusion after initial loading of 6–8 g/3–4 g - Inhaled Colistin: - 2 million units every 8 h
